# Protocol for iterative indirect immunofluorescence imaging of frozen mouse intestinal tissues

**DOI:** 10.1016/j.xpro.2026.104410

**Published:** 2026-03-07

**Authors:** Shuji Matsuguchi, Aiyan Yau, Ayaka Omori, Takaya Abe, Hiroshi Kiyonari, Yumi Konagaya

**Affiliations:** 1 Laboratory for Quantitative Biology of Cell Fate Decision, Center for Biosystems Dynamics Research, RIKEN, 2-2-3, Minatojima-minamimachi, Chuo-ku, Kobe 650-0047, Japan; 2 Laboratory for Animal Resources and Genetic Engineering, Center for Biosystems Dynamics Research, RIKEN, 2-2-3, Minatojima-minamimachi, Chuo-ku, Kobe 650-0047, Japan

**Keywords:** Cell Biology, Molecular Biology, Antibody

## Abstract

Here, we present a protocol for iterative indirect immunofluorescence imaging (4i) of frozen tissues (Cryo-4i). We describe the steps for mouse intestinal sample preparation and antibody elution with standard reagents. Avoiding antibody conjugation or specialized equipment makes this protocol cost-effective and accessible. It requires two days for setup and one day per imaging round; five rounds allow multiplexed detection of 10 markers in a single section. We also detail procedures for using a MATLAB-based pipeline which enables quantitative single-cell analysis.

## Highlights

• Procedures for sample preparation of frozen mouse intestinal tissues
• Steps for iterative immunofluorescence imaging on frozen tissues using antibody elution
• Instructions for image analysis of multiple protein markers at single-cell resolution

## Abstract (editor highlights)

Publisher’s note: Undertaking any experimental protocol requires adherence to local institutional guidelines for laboratory safety and ethics.

## Graphical abstract

**Figure:**
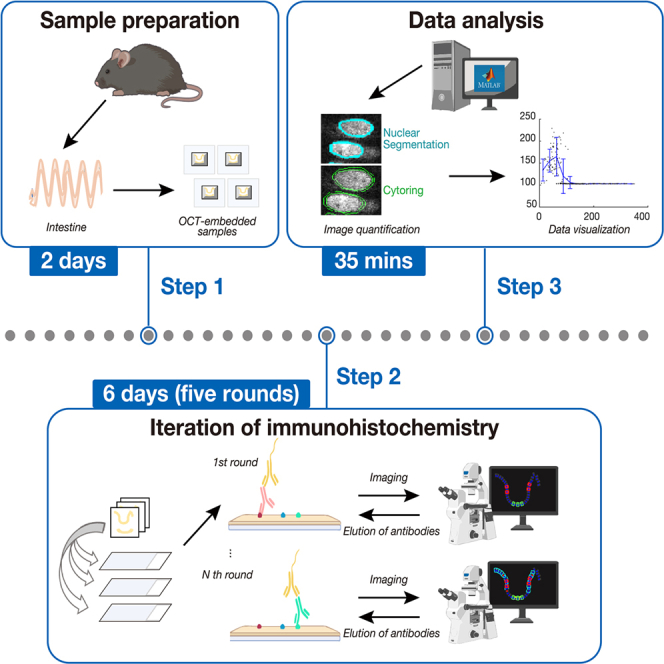

## Before you begin

**Figure 1:**
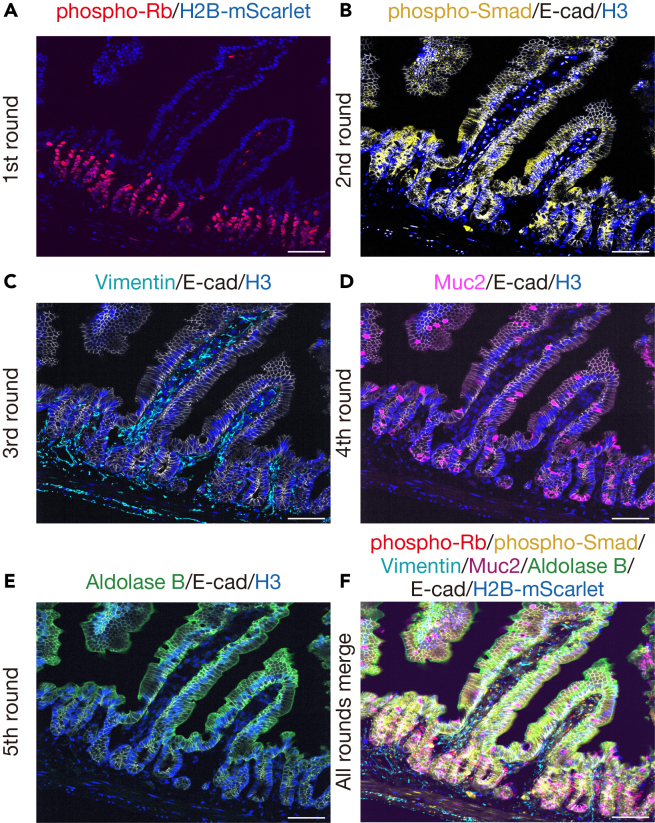
Multiplexed imaging of protein targets from the same tissue sample using Cryo-4i Representative images showing the expression patterns of different protein targets across five rounds of 4i: (A) phospho-Rb (round 1), (B) phospho-Smad1/5/8 (round 2), (C) Vimentin (round 3), (D) Mucin 2 (round 4), (E) Aldolase B (round 5), and (F) merged image of all rounds. All targets were detected using primary antibodies raised in rabbits. For nuclear segmentation, H2B-mScarlet (from a fluorescent reporter mouse) was used in round 1, while Histone H3 and E-cadherin were used from round 2 onwards. Representative data from three biological replicates (n = 3 mice). Scale bars, 50 μm.

Cell signaling plays a critical role in regulating essential biological processes, including development, homeostasis, and disease progression such as cancer.^1^^,^^2^^,^^3^ Advances in multiplexed imaging of protein targets in fixed tissues have significantly enhanced the investigation of such cell signaling networks within tissues.^4^^,^^5^ However, many existing methods depend on antibody conjugation and/or specialized equipment, which can limit accessibility. In contrast, iterative indirect immunofluorescence imaging (4i) provides a powerful approach for acquiring multiplexed spatial protein data through simple iterative elution steps, eliminating the need for both antibody conjugation and specialized equipment.^6^^,^^7^ This accessibility makes the 4i method broadly applicable across molecular biology laboratories. Protocols of 4i have been established for fixed cultured cells, formalin-fixed paraffin-embedded (FFPE) tissue sections, and metaphase chromosome spreads.^8^ Compared to FFPE tissues, frozen tissues allow for faster processing and superior preservation of DNA, RNA, and proteins, making them particularly advantageous for molecular analyses. Therefore, we introduce a protocol of 4i adapted specifically for frozen tissue sections, termed Cryo-4i. This adapted protocol provides detailed instructions and critical considerations for achieving effective staining and gentle handling of tissue samples on the glass slides. First, compared to the original 4i protocol, the primary antibodies are applied overnight at 4°C instead of two hours at room temperature (20∼25°C) to ensure robust and specific staining. Second, the use of imaging buffer supplemented with 50% glycerol, combined with gentle removal of coverslips in a PBS-containing vertical staining jar, reduces possible mechanical shearing stress on the tissue, thereby preserving tissue integrity across multiple rounds of 4i. Finally, we provide a MATLAB-based analysis pipeline that corrects the XY jitter and quantitatively assesses multiple protein targets such as expression levels, localization, and post-translational modifications, at the single-cell level. To demonstrate the utility of Cryo-4i, we applied the protocol to mouse intestinal tissues and managed to detect 10 markers across 5 rounds in a single section (Figures 1A–1F), enabling quantitative analysis of cell signaling and cell cycle states in specific cell types. The Cryo-4i protocol provides a robust approach for obtaining comprehensive and quantitative single-cell data for protein targets while maintaining spatial resolution within tissues, expanding opportunities for a broader range of laboratories to study complex cellular signaling networks.

The Cryo-4i protocol offers broad applicability, being suitable not only for intestinal tissues but also for other tissues such as the brain, muscle, and pancreas, where frozen tissues are commonly used in immunohistochemistry. A caveat of the Cryo-4i protocol is the potential risk of tissue architecture damage across multiple rounds of 4i, particularly during the elution step. Although we have not systematically compared different tissue types, in general, tissues with fragile structures and/or low extracellular matrix content are considered more susceptible to damage, whereas structurally dense tissues are relatively more resistant.

### Innovation

We developed a multicycle immunohistochemistry (IHC) method for frozen tissue sections, termed Cryo-4i, based on the 4i technology.^6^ Frozen tissue sections offer advantages over formalin-fixed paraffin-embedded (FFPE) tissue sections, including better antigen retention and shorter processing times. However, cryosections are prone to detaching from slides, particularly during the antibody elution and coverslip removal steps. To address this issue, we describe technical strategies involving the use of high-adhesion glass slides, imaging in the imaging buffer supplemented with 50% glycerol, and the gentle removal of coverslips in a PBS-containing vertical staining jar. These steps help preserve the structural integrity of fragile frozen tissues across multiple 4i rounds.

Furthermore, we developed an open-source MATLAB-based image analysis pipeline (GitHub: https://github.com/Yumi-Konagaya-Lab/cryo-4i-image-analysis-matsuguchi-2025.git) building on a previously published framework^9^^,^^10^ (GitHub: https://github.com/scappell/Cell_tracking), which was later modified in subsequent studies^11^^,^^12^ (GitHub: https://github.com/MeyerLab/image-analysis-ratnayeke-2022; GitHub: https://github.com/MeyerLab/image-analysis-konagaya-2022). Our new pipeline introduces several key enhancements: (1) Taking E-cadherin staining into account to assist nuclear segmentation, (2) use of E-cadherin signal to distinguish epithelial cells from other cell types, and (3) visualization of data with spatial context, such as the crypt–villus axis.

In recent decades, numerous multiplexed imaging technologies have been developed to analyze proteins in fixed tissues. In contrast to existing methods, the Cryo-4i protocol presented here offers several distinct advantages when applied to frozen tissues. It utilizes only standard molecular biology reagents, avoiding the need for antibody conjugation, expensive reagents, or specialized equipment, thus providing a cost-effective and accessible alternative to other multiplexed imaging techniques.

### Institutional permissions

All animal procedures were conducted in compliance with local ethical guidelines. Mice were maintained and bred under specific pathogen-free (SPF) conditions with approval from the Institutional Animal Care and Use Committee of RIKEN Kobe Branch.

### Reagent preparation

**Timing: 30∼60 min**

Before starting the protocol, be sure to prepare 1% (vol/vol) formaldehyde phosphate buffer solution, 12%/15%/18% (wt/vol) sucrose solution, 0.1% (vol/vol) Triton X-100 solution, 1% (wt/vol) BSA solution, imaging buffer (50% glycerol (vol/vol) in PBS), and elution buffer according to the Materials section. It is also important to make sure you have other reagents including 70% ethanol, PBS (ice-cold and room temperature), O.C.T. compound, primary and secondary antibodies, Hoechst, and antigen retrieval reagents such as HistoVT One.

## Key resources table

**Table:** 

REAGENT or RESOURCE	SOURCE	IDENTIFIER
**Antibodies**		

Phospho-Rb (Ser807/811) (D20B12) (dilution 1:2500)	Cell signaling Technology	Cat#8516S; RRID:AB_11178658
Phospho-Smad1/5/8 (Ser463/465) (dilution 1:50)	Merck	Cat#AB3848-I; RRID:AB_177439
Vimentin (dilution 1:500)	Cell signaling Technology	Cat#5741T; RRID:AB_10695459
E-cadherin (dilution 1:1)	This paper	Generated from the ECCD2 hybridoma
Muc2 (dilution 1:500)	Abcam	Cat#ab272692; RRID:AB_2888616
Aldolase B (dilution 1:500)	Abcam	Cat#ab75751; RRID:AB_2226682
Histone H3 (D1H2) XP® Rabbit mAb (Alexa Fluor® 647 Conjugate) (dilution 1:100)	Cell signaling Technology	Cat#12230S; RRID:AB_2797852
Rabbit IgG (H+L) Highly Cross-Adsorbed Secondary Antibody, Alexa Fluor™ 488 (dilution 1:2000)	Thermo Fisher	Cat#A-11034; RRID:AB_2576217
Rat IgG (H+L) Cross-Adsorbed Secondary Antibody, Alexa Fluor™ 568 (dilution 1:2000)	Thermo Fisher	Cat#A-11077; RRID:AB_2534121
Rabbit IgG (H+L) Highly Cross-Adsorbed Secondary Antibody, Alexa Fluor™ 647 (dilution 1:2000)	Thermo Fisher	Cat#A-21245; RRID:AB_2535813

**Chemicals, peptides, and recombinant proteins**		

4%-Paraformaldehyde Phosphate Buffer Solution	Nacalai tesque	Cat#09154-85
HistoVT One (10x) (pH 7.0)	Nacalai tesque	Cat#06380-05
Triton X-100	Nacalai tesque	Cat#35501-15
Tissue-Tek O.C.T. compound	Sakura Finetek Japan	Cat#4583
Hoechst 33342	Thermo Fisher	Cat#H3570
Sucrose	Nacalai tesque	Cat#30404-45
Bovine Serum Albumin (BSA)	Nacalai tesque	Cat#01860-07
L-glycine	Nacalai tesque	Cat#17141-95
Urea	Nacalai tesque	Cat#35905-35
Guanidine Hydrochloride	Nacalai tesque	Cat#17318-95
Tris(2-carboxyethyl) phosphine hydrochloride (TCEP-HCl)	Nacalai tesque	Cat#07277-16
Phosphate Buffered Saline (10x) (pH 7.4)	Nacalai tesque	Cat#27575-31
Glycerol	Nacalai tesque	Cat#17017-35

**Deposited data**		

Raw Cryo-4i Image Data	This paper	https://doi.org/10.24631/ssbd.repos.2026.02.491

**Experimental models: Cell lines**		

Hybridoma cells: ECCD-2 cells	RIKEN BioResource Research Center (BRC)	ECCD-2: RIKEN BRC #RCB5259; RRID:CVCL_A6WX

**Experimental models: Organisms/strains**		

Mouse: R26R-H2B-mScarlet (adult homozygous C57BL/6 male and female mice for breeding)	This paper; RIEKN Center for Biosystems Dynamics Research (BDR) Laboratory for Animal Resources and Genetic Engineering	#CDB407E
Mouse: R26-H2B-mScarlet (adult homozygous C57BL/6 male and female mice for breeding)	This paper; RIEKN Center for Biosystems Dynamics Research (BDR) Laboratory for Animal Resources and Genetic Engineering	#CDB408E
Mouse: LGR5-eGFP-IRES-CreERT2 (adult heterozygous C57BL/6 male and female mice for breeding)	The Jackson Laboratory	#JAX008875; RRID:IMSR_JAX:008875
Mouse: R26-H2B-mScarlet; LGR5-eGFP-IRES-CreERT2 (9–12-week-old heterozygous C57BL/6 male mice for data collection)	This paper	Crossing Mouse #CDB408E and #JAX008875

**Software and algorithms**		

MATLAB	MathWorks	https://www.mathworks.com/products/matlab.html
BioRender	BioRender	https://www.biorender.com/
Cryo-4i Image Analysis Pipeline	This paper; GitHub	https://github.com/Yumi-Konagaya-Lab/cryo-4i-image-analysis-matsuguchi-2025.git

**Other**		

Cryostar NX70	Thermo Fisher	Cat#957030
Microtome blade	FEATHER Safety Razor	Cat#C35
Fisherbrand™ Superfrost Plus™ Microscope Slides	Fisher scientific	Cat#12-550-15
Micro cover glass 24×60 No.1	Matsunami Glass	Cat#C024601
Tissue-Tek Cryomold	Sakura Finetek Japan	Cat#4566
Tissue-Tek Slide Warmer	Sakura Finetek Japan	Cat#PS-53
Invitro shaker	TAITEC	Shake-LR
10 cm dish	Greiner	Cat#664161
1.5 ml centrifuge tube	Bio-BIK	Cat#LT-0150
15 ml centrifuge tube	WATSON	Cat#1332-015S
50 ml centrifuge tube	FALCON	Cat#352070
100 ml centrifuge tube	IWAKI	Cat#2355-100
Dragonfly 200, Inverted confocal microscope	Andor/Olympus	N/A
UPLSAPO30XS	Olympus (1.05 NA)	N/A

## Materials and equipment

**Table:** 1% (vol/vol) formaldehyde phosphate buffer solution

Reagent	Final concentration	Amount
4% Paraformaldehyde Phosphate Buffer Solution (PFA)	1%	3 mL
Phosphate-buffered saline (PBS)	N/A	9 mL
**Total**	**N/A**	**12 mL**

Store at 4°C for up to 1 week.

**Table:** 12%/15%/18% (wt/vol) sucrose solution

Reagent	Final concentration	Amount
Sucrose	12%, 15%, or 18%	12 g, 15g, or 18g
Phosphate-buffered saline (PBS)	N/A	100 mL
**Total**	**N/A**	**100 mL**

Store at 4°C for up to 1 month.

**Table:** 0.1% (vol/vol) Triton X-100 solution

Reagent	Final concentration	Amount
Triton X-100	0.1%	10 μL
Phosphate-buffered saline (PBS)	N/A	9.99 mL
**Total**	**N/A**	**10 mL**

Store at 4°C for up to 1 month.

**Table:** 1% (wt/vol) BSA solution

Reagent	Final concentration	Amount
Bovine serum albumin (BSA)	1%	1 g
Phosphate-buffered saline (PBS)	N/A	100 mL
**Total**	**N/A**	**100 mL**

Store at 4°C for up to 1 month.

**Table:** Imaging buffer (50% glycerol (vol/vol) in PBS)

Reagent	Final concentration	Amount
Glycerol	50%	5 mL
Phosphate-buffered saline (PBS)	N/A	5 mL
**Total**	**N/A**	**10 mL**

As in the original 4i protocol, 700 mM N-acetyl cysteine can be added, and the solution adjusted to pH 7.4 with NaOH. N-acetyl cysteine in the imaging buffer scavenges free radicals generated during light exposure, helping to prevent the formation of photoreactive crosslinks. Store at 4°C for up to 1 month.

**Table:** Elution buffer

Reagent	Final concentration	Amount
L-glycine	500 mM	11.25 g
Urea	3 M	54 g
Guanidinium hydrochloride	3 M	86 g
Tris(2-carboxyethyl) phosphine hydrochloride (TCEP-HCl)	70 mM	6 g
3 N HCl	(to pH 2.5)	X mL
Double-distilled water (ddW)	N/A	Complete volume to 300 mL
**Total**	**N/A**	**300 mL**

Depending on the primary antibodies, add 1/100 volume of 2-mercaptoethanol and mix well just before use. As a general rule, addition of 2-mercaptoethanol to the elution buffer is recommended only when the staining remains after elution. Store at 4°C for up to 1 month.

## Step-by-step method details

### Dissection and mounting of mouse intestinal tissue

**Timing: ∼7.5 h (∼30 min dissection /mice + ∼7 h tissue mounting for cryosectioning)**

This section describes isolation of the mouse small intestine and prepares it for cryosectioning. It preserves tissue structure and antigens through careful dissection, fixation, cryoprotectant infiltration, and embedding in O.C.T. compound.

1. Euthanize the mouse following animal ethical guidelines.

***Note:*** This protocol has been developed using young (9–12 weeks old) male mouse intestinal tissue.

2. Place the euthanized mouse in a supine position and secure it to a dissection board using pins.
3. Position the mouse and expose the abdominal area for dissection.
  a. Moisten the abdominal area with 70% ethanol.
  b. Make a small incision in the abdominal wall using dissecting scissors.
  c. Carefully extend the incision from the abdomen to the chest.
4. Access the abdominal cavity to isolate the small intestine.
  a. Once the abdominal cavity is exposed, locate the stomach by gently separating the surrounding connective tissue to avoid damage.
  b. Cut at the junction between the stomach and duodenum using dissecting scissors.
  c. Carefully remove the small intestine from the body.
5. Isolate the small intestinal region (duodenum, jejunum, or ileum) from the mouse. To isolate duodenum, harvest approximately 7 cm from the stomach.
6. Clean the isolated intestine.
  a. Gently rinse the isolated intestine with 10–20 mL ice-cold PBS in a 10 cm dish.
  b. Remove adipose tissues as thoroughly as possible.
7. Carefully open the duodenum longitudinally using dissecting scissors to expose the luminal surface.
8. Rinse the lumen with 10–20 mL of ice-cold PBS in a 10 cm dish to remove residual intestinal contents.

**CRITICAL:** If working with tissues expressing fluorescent proteins from reporter mice, all subsequent steps are performed under light-shielded conditions to preserve fluorescence.

9. Wash and fix the isolated intestinal tissue.
  a. After washing with PBS, fix both ends of the tissue onto a small piece of heavy paper using a staple.
  b. Place the sample in a 15 mL tube containing 1% PFA in PBS.
  c. Incubate the sample at 4°C overnight (∼20 h).

**CRITICAL:** Immerse the isolated small intestine in 1% PFA in PBS as soon as possible because the intestine contains abundant proteases and peptidases that could degrade or denature sample antigens.

***Note:*** Tissue fixation in a 15 mL tube using a shaker or rotor is not recommended, as the turbulence can damage the villi structures.

10. Perform cryoprotectant infiltration of the fixed tissues.
  a. Immerse the fixed tissues in 12% sucrose in PBS at 4°C for 2 h.
  b. Transfer the tissues to 15% sucrose in PBS at 4°C for 2 h.
  c. Transfer the tissues to 18% sucrose in PBS at 4°C for 2 h.

***Note:*** Cryoprotectant infiltration in a 15 mL tube using a shaker or rotor is not recommended, as the turbulence can damage the villi structures.

11. Embed the tissues in O.C.T. blocks.
  a. Cut the intestine into segments approximately 5 mm to 1 cm in length.
  b. Embed them in O.C.T. compound for cryosectioning.
  c. Orient each tissue segment so that the cross-sectional surface faces the cutting surface of the O.C.T. block.
12. Snap-freeze all O.C.T.-embedded samples by placing them in a metal container submerged in liquid nitrogen and immediately store the frozen samples at −80°C.

**Pause point:** At this stage, fixed tissues including those expressing fluorescent proteins (e.g., from reporter mice) can be stored at −80°C for up to 2–3 months.

### 4i staining workflow: Initial staining, image acquisition, antibody elution, and iteration

**Timing: 2∼6 days (two days for setup and one day per imaging round)**

**Figure 2:**
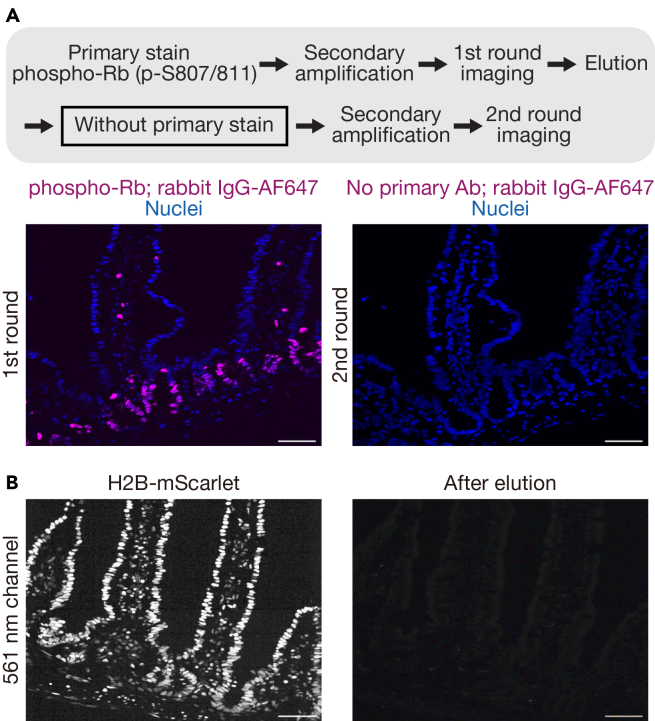
Validation of antibody elution in Cryo-4i (A) Schematic diagram outlining the workflow for elution validation (top). As a negative control, the standard Cryo-4i protocol was performed, except that in the second round, the tissue was re-stained with secondary antibody only, without any primary antibody. Tissue staining for phospho-Rb (magenta) and nuclei visualized with H2B-mScarlet (blue) from the first round of 4i is shown (bottom). No detectable fluorescence signal in the 647 nm channel was observed in the second round, indicating sufficient antibody elution. Scale bars, 50 μm. (B) H2B-mScarlet fluorescence from the reporter mouse was detectable in the first round (left), but not in the second round (right), indicating that the fluorescence of the reporter proteins is lost after the elution step.

**Figure 3:**
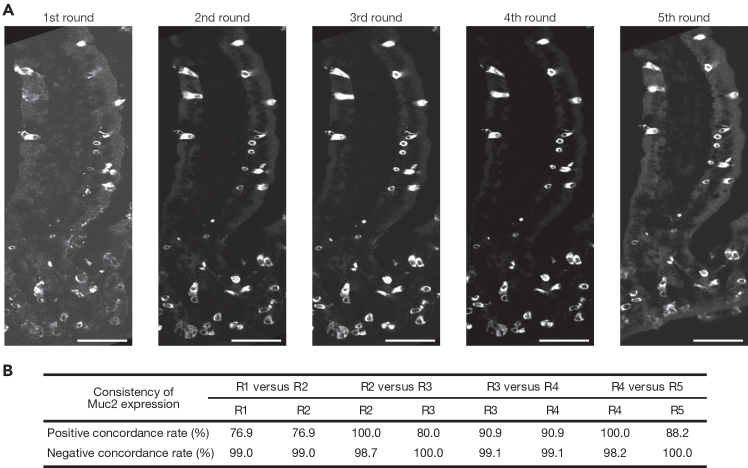
Validation of consistent tissue staining across multiple Cryo-4i rounds (A) Representative images of mouse intestinal epithelium stained for Mucin 2 across all five rounds of 4i. Representative data from three biological replicates (n = 3 mice). Scale bars, 50 μm. (B) Table showing the positive and negative concordance rates of Mucin 2 expression between the Nth and (N+1)th rounds of 4i. The high concordance across rounds confirms the reproducibility of tissue staining throughout multiple Cryo-4i cycles. Representative data from three biological replicates (n = 3 mice). Scale bars, 50 μm.

This section describes iterative indirect immunofluorescence imaging (4i) on frozen tissue sections, allowing multiple protein markers to be visualized sequentially on the same sample. It includes initial staining, imaging, antibody elution, and repeated rounds, preserving tissue structure and antigenicity for multiplexed analysis (as validated in Figures 2 and 3).

13. Equilibrate the frozen samples in the cryostat for at least 15 min.
14. Section frozen tissue at 7 μm and mount onto the glass slide.

***Note:*** We have tested thicker sections of 10 μm and did not encounter any issues. Sections thicker than 10 μm have not been tested and may require further optimization.

**CRITICAL:** To avoid tissue detachment from the glass slides, it is critical to use high-adhesion glass slides (e.g., Fisherbrand™ Superfrost Plus™ Microscope Slides).

15. Prepare the tissue sections for staining.
  a. Air-dry the tissue sections for at least 10 min.
  b. Wash off the O.C.T. compound with PBS once for 10 min on a shaker.

**CRITICAL:** Tissue sections cannot be stored again at −80°C because epitope integrity and fluorescence of the reporter proteins can be compromised under these conditions.

16. Post-fix the tissue sections on the glass slide.
  a. Fix the tissue sections with 4% PFA in PBS for 10 min at room temperature (20°C∼25°C).
  b. Wash the tissue sections three times with PBS for 10 min each on a shaker.

**CRITICAL:** It is critical to perform this post-fixation to avoid tissue detachment from the glass slides.

Troubleshooting 1.

17. Permeabilize the tissue sections.
  a. Permeabilize the tissue sections using 0.1% Triton X-100 in PBS for 10 min at room temperature (20°C∼25°C).
  b. Wash the tissue sections three times with PBS for 10 min each on a shaker.
18. Block the tissue sections with 1% (wt/vol) BSA solution for 60 min at room temperature (20°C∼25°C).
19. Apply the primary antibody diluted in BSA solution to the tissue sections (see key resources table for antibody concentrations). When not using a rat-derived primary antibody, add the rat-derived E-cadherin primary antibody to the same incubation solution.

**CRITICAL:** As a general rule, perform the first round of 4i with antibodies that stain weakly (e.g., highly sensitive antigens and protein modifications) and use antibodies that produce strong staining in later rounds of 4i.

20. Place the slides in a humidified chamber and incubate overnight (∼16 h) at 4°C.
21. Wash the tissue sections three times with PBS for 10 min each on a shaker to remove the primary antibody.
22. Apply the secondary antibody (e.g., Rat IgG (H+L) Cross-Adsorbed Secondary Antibody, Alexa Fluor™ 568 and Rabbit IgG (H+L) Highly Cross-Adsorbed Secondary Antibody, Alexa Fluor™ 647) diluted 1: 2000 in BSA solution for 60 min at room temperature (20°C∼25°C).

**CRITICAL:** All subsequent steps are performed under light-shielded conditions to preserve fluorescence from the secondary antibodies.

23. Wash the tissue sections three times with PBS for 10 min each on a shaker to remove the secondary antibody.
24. Apply Hoechst in PBS (1:5000; 2 μg/mL) for 15 min at room temperature (20°C∼25°C).
25. Wash the tissue sections three times with PBS for 10 min each on a shaker to remove Hoechst.
26. Mount the tissue sections in imaging buffer.
  a. Add 180 μL of imaging buffer dropwise onto the slide.
  b. Carefully place the coverslip over the tissue section to avoid air bubbles.

**CRITICAL:** Do not seal the coverslip to the slide, as it needs to be removed after imaging for subsequent rounds of 4i.

27. Acquire images of the tissue sections using an inverted confocal microscope equipped with a medium-magnification immersion objective lens (20x-40x).

**CRITICAL:** During imaging, manually record the X and Y coordinates of the field of view or ensure that the image acquisition software saves this information in the image properties. These coordinates are essential for relocating the same area in the subsequent rounds of 4i. Additionally, capturing overview images using a low-magnification objective lens (e.g., 4x-10x) facilitates navigation across rounds.

***Note:*** We perform fluorescence imaging using an Andor Dragonfly 200 inverted confocal microscope equipped with an Olympus UPLSAPO30XS (30x silicone-immersion) objective lens and an Andor Zyla Z4.2P-USB3 sCMOS camera. Laser power is typically set to ∼10%, and camera exposure time to ∼100 ms (Figure S1). Detailed microscope specifications and image acquisition parameters are provided in the Microscopy Metadata Checklist (Methods S1). Although wide-field microscopes equipped with dry objectives can be used, confocal microscopes are recommended for higher resolution. Furthermore, while water- or oil-immersion objectives are compatible, silicone-immersion objectives are preferred because their refractive index (∼1.4) closely matches that of the imaging buffer (50% glycerol/PBS).

Troubleshooting 2.

28. After imaging, remove the coverslip from the slide.
  a. Place the slide in a PBS-containing vertical staining jar.
  b. Use a pipette tip or tweezers to assist gentle removal of coverslip in the jar.
  c. Wash off the imaging buffer twice with PBS for 10 min each on a shaker.

**CRITICAL:** When removing the coverslip, handle it gently so that it comes off by its own weight while remaining fully submerged in PBS. This helps prevent tissue tearing or detachment.

**Pause point:** The samples can be stored for a week at 4°C by immersing them in PBS to prevent drying.

29. Apply the elution buffer to the tissue section at room temperature (20°C∼25°C), repeat three times, replacing the elution buffer every 10 min.

**CRITICAL:** Frozen sections are more fragile than FFPE sections. Using high-adhesion glass slides (e.g., Fisherbrand™ Superfrost Plus™ Microscope Slides) helps maintain tissue integrity.

**CRITICAL:** For primary antibody not listed in key resources table, elution buffer composition and condition may need to be optimized based on multiple factors, including the expression level of the target protein, its stability, and the binding affinity of the primary antibody to its target. Less abundant/unstable proteins and/or lower-affinity antibodies can be eluted using a mild elution protocol, whereas highly expressed/stable proteins and/or higher-affinity antibodies may require harsh elution conditions within the range that preserves tissue integrity.

Another factor to be considered is the properties specific to subsequent staining. For example, if the subsequent primary antibody yields weak or no signal, milder elution conditions may be needed to preserve epitope integrity. For milder elution, consider the following adjustments: reducing the concentration of protein reductant (TCEP) and denaturants (urea and guanidine hydrochloride) and/or shortening the incubation time.

Conversely, if residual staining from the previous primary antibody is detected, consider testing harsher elution conditions within the range that preserves the tissue integrity. To confirm the effectiveness of the elution step, first simply observe the sample again after elution under microscope. Then, omit the primary antibody for the second round and apply only the secondary antibody solution for 60 min at room temperature (20∼25°C), followed by Hoechst staining (example shown in Figure 2). If any primary antibody from the previous round remains, it will be detected by the secondary antibodies in this negative control, indicating insufficient elution. In such cases, consider the following adjustments to achieve harsher elution conditions: increasing the concentration of protein reductant (TCEP) and denaturants (urea and guanidine hydrochloride), raising the reaction temperature (e.g., 55°C), or extending the incubation time to ensure more thorough removal of the bound antibody. Another reductant, 2-mercaptoethanol is also used for antibody elution in a different multiplexed immunohistochemistry method, Multiple Iterative Labeling by Antibody Neodeposition (MILAN).^13^^,^^14^ However, repeated elution with 2-mercaptoethanol (e.g., over five rounds of 4i) can compromise epitope stability and reduce the staining signal-to-noise ratio. It is therefore recommended to minimize the use of 2-mercaptoethanol whenever possible.

Troubleshooting 3 and 4.

30. After the elution step, wash off the elution buffer three times with PBS for 10 min each on a shaker.

**Pause point:** The samples can be stored for several days at 4°C by immersing them in PBS to prevent drying.

31. Block the tissue sections with 1% (wt/vol) BSA solution for 60 min at room temperature (20∼25°C).

**CRITICAL:** If antigen retrieval is necessary, it should be performed using HistoVT One (which contains citrate buffer) or another suitable reagent (e.g., citrate buffer) before the blocking step.

32. Apply the primary antibody diluted in BSA solution to the tissue sections (see key resources table for antibody concentrations). When not using a rat-derived primary antibody, add the rat-derived E-cadherin primary antibody to the same incubation solution.

***Note:*** See Step 19 for important cautions regarding antibody prioritization.

33. Place the slides in a humidified chamber and incubate overnight (∼16 h) at 4°C.
34. Wash the tissue sections with PBS to remove the primary antibody.
35. Apply the secondary antibody (e.g., Rabbit IgG (H+L) Highly Cross-Adsorbed Secondary Antibody, Alexa Fluor™ 488 and Rat IgG (H+L) Cross-Adsorbed Secondary Antibody, Alexa Fluor™ 568) diluted 1:2000 in BSA solution for 60 min at room temperature.
36. Wash the tissue sections with PBS to remove the secondary antibody.
37. Apply the conjugated primary antibody (Histone H3 (D1H2) XP® Rabbit mAb (Alexa Fluor® 647 Conjugate)) diluted in BSA solution for the Histone H3 protein staining for 2 h at room temperature (20∼25°C).
38. Wash the tissue sections with PBS to remove the conjugated primary antibody.
39. Apply Hoechst in PBS (1:5000; 2 μg/mL) for 15 min at room temperature (20∼25°C).
40. Wash the tissue sections with PBS to remove Hoechst.
41. Mount the tissue sections in imaging buffer.
  a. Add 180 μL of imaging buffer dropwise onto the slide.
  b. Carefully place the coverslip over the tissue section to avoid air bubbles.

**CRITICAL:** Do not seal the coverslip to the slide, as it needs to be removed after imaging for subsequent rounds of 4i.

42. Acquire images of the tissue sections using the same inverted confocal microscope as in Step 27 to maintain the identical image acquisition settings.

**CRITICAL:** During imaging, capture overview images using a low-magnification objective lens and record the X and Y coordinates of the field of view, as these are essential for relocating the same area in the subsequent rounds of 4i, as described in Step 25 previously. In addition, a direct comparison of the images captured during the first round can aid in accurate relocation of the field of view.

43. After imaging, remove the coverslip from the slide.
  a. Place the slide in a PBS-containing vertical staining jar.
  b. Use a pipette tip or tweezers to assist gentle removal of coverslip in the jar.
  c. Wash off the imaging buffer with PBS.

**Pause point:** The samples can be stored for several days at 4°C by immersing them in PBS to prevent drying.

44. If you plan to continue with the iterative staining protocol, repeat the procedure starting from Step 26.

### Image processing and quantification in MATLAB

**Timing: ∼30 min**

**Figure 4:**
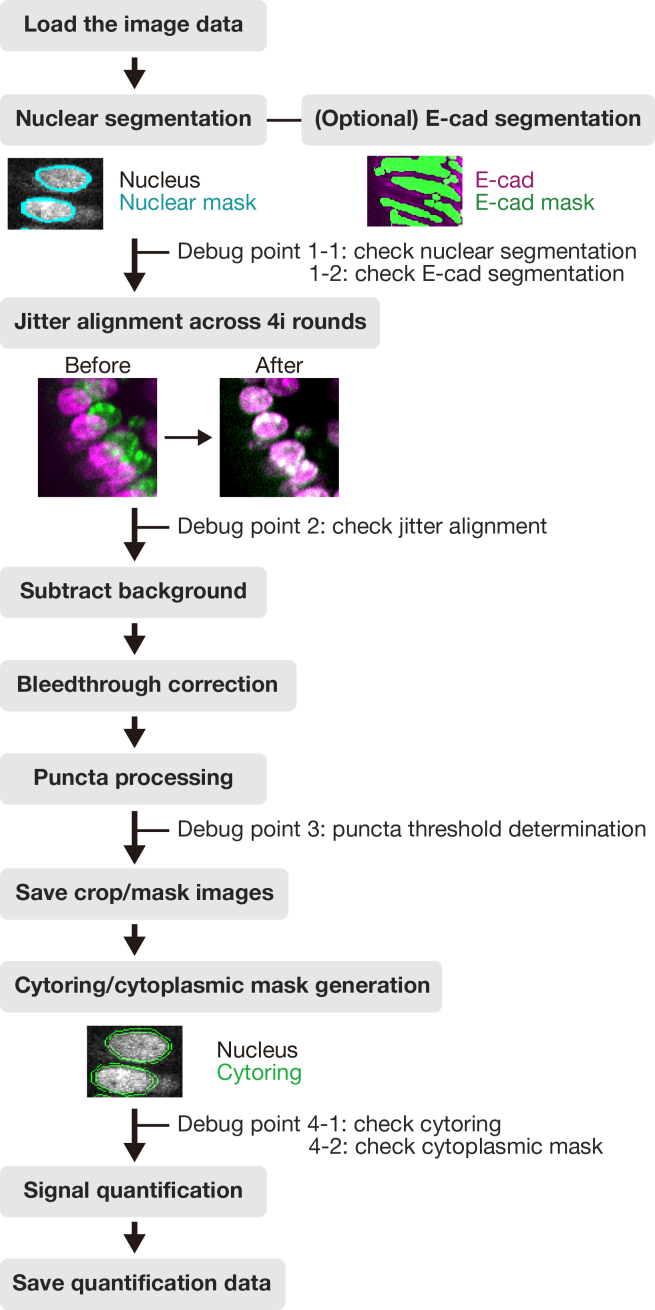
Schematic illustration of image processing and quantification in MATLAB The diagram outlines the workflow of the MATLAB-based image processing pipeline. Key processing steps are shown, along with debug points illustrating the expected appearance of the representative debug images.

**Figure 5:**
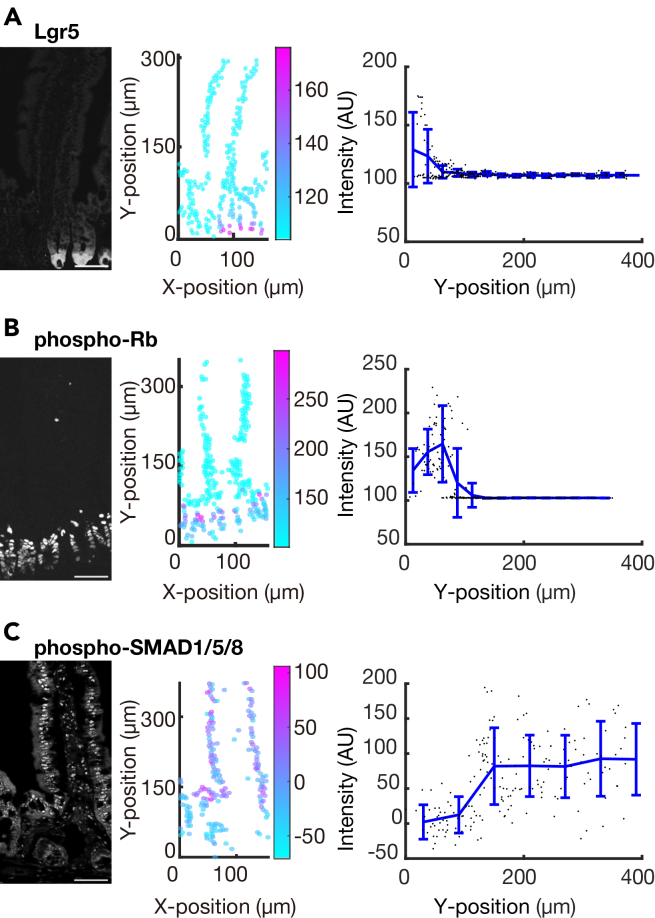
Quantification of protein signals in mouse small intestinal epithelium in the MATLAB-based image processing pipeline The representative images (left), corresponding quantification color-coded by the protein of interest (POI) intensity at the single-cell level (middle), and quantification of POI intensity as a function of cell position along the crypt-villus axis (right), shown with mean + standard deviation (SD). (A) Lgr5 intensity in the cytoring. (B) phospho-Rb intensity in the nucleus. (C) phospho-Smad1/5/8 intensity in the nucleus minus the cytoring. Representative data from three biological replicates (n = 3 mice). Scale bars, 50 μm.

This section describes the process of converting raw Cryo-4i images into analyzable data using MATLAB. It includes nuclear and cell segmentation, alignment across rounds, signal extraction, and generation of quantitative outputs for downstream visualization and analysis (Figures 4 and 5).

45. Convert the raw microscopy images to a MATLAB-compatible file format (e.g., TIFF) for import. Preserve multi-channel images as single multi-stack files.

***Note:*** There is no strict file size limit for MATLAB scripts or function files. MATLAB can execute.m files of virtually any size as long as sufficient system memory is available. The code can handle both 12-bit and 16-bit images, as MATLAB internally stores these as 16-bit integers (uint16).

46. Use the same file name for 4i images from different rounds, and save each round in a separate subfolder within the same parent directory. Each file name must include the site (field of view) number at the end (e.g., example_1.tif).
47. Set the input settings parameters in the MATLAB script run_cryo4i.m.

***Note:*** The parameters are described as follows:

file_name: File name of the raw microscopy image.

data_path: Output folder path for MATLAB data file.

IF_imagesessions: Input folder path for the raw microscopy images. For multi-round imaging, list the folders separated by commas (,).

bgcmospath: Folder path for background images used with CMOS camera detection. Leave blank (‘’) if no background images are used.

crop_save: Output folder path for the cropped images after jitter alignment across 4i rounds.

mask_save: Output folder path for the nuclear mask images.

maskname: Name assigned to mask images (e.g., ‘mask’).

primaryMaskRound: The reference round for comparing images across 4i rounds.

maskIndex: The nuclear channel number for nuclear segmentation. Set the channel number individually for each 4i round.

EcadIndex: The E-cadherin channel number for cell segmentation. Set the channel number individually for each 4i round. If no E-cadherin channel is available, set the value to 0.

jitterIndex: The channel number for jitter alignment across 4i rounds. Set the channel number individually for each 4i round.

outerrad_cytoring: The pixel number defining the diameter of the *outer* cytoring, representing the distance extending outward from the nuclear mask. For example, a value of *5* is recommended for the images acquired with x10-x30 objective lenses.

innerrad_cytoring: The pixel number defining the diameter of the *inner* cytoring, representing the distance extending outward from the nuclear mask. For example, a value of *1* is recommended for the images acquired with x10-x30 objective lenses.

postbin: 0 for no software bin, number for scaling factor (e.g., 0.5 for 2x2 bin). Set it individually for each 4i round.

signals: The channel name in each multi-stack microscopy image. Set the channel name individually for each channel and each 4i round.

maxjit: Maximum number of pixels allowed for alignment jitter across 4i rounds. If this limit is exceeded, an error message 'Max jitter too high' will be displayed.

sigblur: The radius in pixels of the circular averaging filter for image blurring. Set the pixel number individually for each channel and each 4i round.

signal_foreground: 1 to enable generation of a mask that includes foreground signal in addition to the nuclear signal for background subtraction, while 0 to disable it. Set to 1 or 0 individually for each channel and each 4i round.

bleedthrough: 1 to enable bleed-through calculation, while 0 to disable it. Set to 1 or 0 individually for each channel and each 4i round.

bleedthroughslope: Scale factor representing the amount of bleed-through from one channel into another. Set this factor individually for each channel pair and each 4i round.

bleedthroughoff: Residual fluorescence intensity remaining after bleed-through correction using the scale factor. Set this value individually for each channel pair and each 4i round.

ringcalc: 1 to enable cytoring calculation, while 0 to disable it. Set to 1 or 0 individually for each channel and each 4i round.

ringthresh: Minimum intensity threshold for effective cytoring used in quantification. Set to 0 for no thresholding. Set to 0 to disable thresholding. Set the number individually for each channel and each 4i round.

punctacalc: 1 to enable puncta calculation (e.g., γ-H2AX signal), while 0 to disable it. Set to 1 or 0 individually for each channel and each 4i round.

punctaThresh: Minimum intensity threshold for detecting effective puncta in the top-hat filtered image. Determine this value based on the average intensity observed in the top-hat filtered image displayed during debug_mode, under the section %% Puncta processing. Set the number individually for each channel and each 4i round.

punctatopsize: The radius in pixels of the top-hat filter used for puncta detection.

cytopuncta: 1 to enable puncta calculation in the cytoplasm, or 0 to disable it and perform puncta calculation in the nucleus. Applicable only when punctacalc is set to 1. Set to 1 or 0 individually for each channel and each 4i round.

thickenradius: The average cytoplasmic radius in pixels in microscopy images. Applicable only when both punctacalc and cytopuncta are set to 1. The default value is typically two to four times larger than nucr.

localbg: 1 to enable local background calculation within a box surrounding each cell, while 0 to disable it. Set to 1 or 0 individually for each channel and each 4i round.

minringsize: Minimum number of pixels for the effective cytoring used in quantification.

bgcmoscorrection: 1 to enable background subtraction within a box surrounding each cell, while 0 to disable it. Applicable only when bgcmospath is defined.

bgsubmethod: Depending on the signal type in each channel, choose the appropriate background subtraction method below:

 ‘global nuclear’ – Suitable for microscopy images with nuclear signals, as it uses the nuclear mask for background subtraction.

 ‘global cyto’ – Suitable for microscopy images with cytoplasmic signals, as it uses cytoplasmic mask for background subtraction.

 ‘tophat’ – Suitable for removing global background (e.g., uneven illumination) in microscopy images, as it uses a top-hat filter for background subtraction.

 ‘semi-local nuclear’ – Suitable for removing semi-local background in microscopy images, as it uses an 11-by-11 block filter for background subtraction.

 ‘none’ – No background subtraction.

Set the background subtraction method individually for each channel and each 4i round.

bgperctile: The percentile number of pixel intensities in the masked/filtered image for background subtraction. Applicable only when bgsubmethod is ‘global nuclear’, ‘global cyto’, or ‘semi-local nuclear’. Set the percentile number individually for each channel and each 4i round. For example, a value of 25 is recommended for our microscopy images.

segmethod: Depending on the signal characteristics in each nuclear channel, choose the appropriate nuclear segmentation method below:

 ‘log’ – Suitable for detecting non-circular nuclei. Nuclear segmentation using log-scaled nuclear images.

 ‘thresh’ – Nuclear segmentation after global image thresholding using Otsu’s method.

 ‘single’ – Nuclear segmentation after global image thresholding using Otsu’s method followed by watershed transformation.

 ‘double’ –Nuclear segmentation after global image thresholding using Otsu’s method followed by watershed transformation, and a second Otsu thresholding for high-intensity signals.

 ‘double marker’ – Nuclear segmentation after global image thresholding using Otsu’s method followed a second Otsu thresholding for high-intensity signals, and watershed transformation.

 ‘multithresh’ – Nuclear segmentation after multilevel image thresholding using Otsu’s method followed by watershed transformation.

 ‘concavity’ – Nuclear segmentation using a threshold determined by identifying the most convex and concave point in the intensity distribution.

 ‘log contour’ – Suitable for detecting circular nuclei. Nuclear segmentation using active contours (snakes) region growing technique.

 ‘Nuc-Ecad boundary’ – Suitable for detecting concatenated nuclei with E-cadherin boundaries. Nuclear segmentation using the ‘thresh’ method followed by concatenated nuclei separation based on E-cadherin signals.

 ‘Ecad boundary’ –Suitable for detecting cells that lack strong or detectable nuclear but exhibit E-cadherin signals. Cell segmentation using only E-cadherin signals; therefore, in the final output, the object centroid and area correspond to the cell, not the nucleus.

nucr: The average nuclear radius in pixels in microscopy images.

debrisarea: Minimum number of pixels for the effective nuclear masks used in quantification. Set the threshold number individually for each 4i round.

boulderarea: Maximum number of pixels for the effective nuclear masks used in quantification.

blobthreshold: Threshold number for nuclear segmentation. Applicable only when segmethod is set to ‘log’ or ‘log contour’. Higher values tend to result in the detection of more debris, whereas lower values may lead to incomplete detection of nuclei. The default value is −0.01 to −0.03.

blurradius: The radius in pixels of the circular averaging filter for nuclear segmentation. Applicable only when segmethod is set to ‘thresh’, ‘single’, ‘double’, ‘double marker’, ‘multithresh’, ‘concavity’ or ‘Nuc-Ecad boundary’. The default value is 3.

soliditythresh: Threshold number for nuclear solidity. Solidity is calculated by dividing the contour area by the convex hull area. The default value is 0.3 to 0.8.

compression: The scale factor in compression for background subtraction. Applicable only when segmethod is set to ‘global nuclear’, ‘global cyto’, or ‘semi-local nuclear’. The default value is 4.

split_mult: Threshold number for concatenated nuclei separation. The default value is 1.

Edge_method: Depending on the signal characteristics in each E-cadherin channel, choose the appropriate Edge detection method below:

 ‘log’ – Edge detection by identifying zero-crossings after applying a Laplacian of Gaussian (LoG) filter.

 ‘Canny’ – Edge detection by identifying local maxima in the gradient of the image. The edge function calculates the gradient using the derivative of a Gaussian filter. This method uses two thresholds to detect strong and weak edges, including weak edges in the output if they are connected to strong edges. By using two thresholds, the Canny method is less susceptible to noise and more effective at detecting true weak edges.

The default method is ‘Canny’.

R_strel_edge: The radius in pixels of the circular averaging filter to close the gap in E-cadherin edges.

R_strel_extended: The radius in pixels of the circular averaging filter to fill the entire E-cadherin positive cell area.

Pixel_connectivity: The pixel connectivity to fill holes in the filtered E-cadherin image. Accepted values are 4 or 8, with a default value of 4.

Max_pixels: Minimum number of pixels for the effective E-cadherin masks used in cell segmentation. Applicable only when bgsubmethod is 'Ecad boundary'.

Gaus_filter_sigma: Standard deviation value of a Gaussian smoothing kernel to reduce noise in E-cadherin images.

48. To verify the quality of image processing, check the first microscopy image by executing the following command in the MATLAB command window, where site = 1 and debug_mode = 1 (the second and third parameters, respectively):

>Fixed_IF_Ecad(settings,1,1)

49. The MATLAB function Fixed_IF_Ecad will pause at the keyboard command to check the quality of image processing. At that point, check whether the results are satisfactory, then click Continue to proceed.

**Figure 6:**
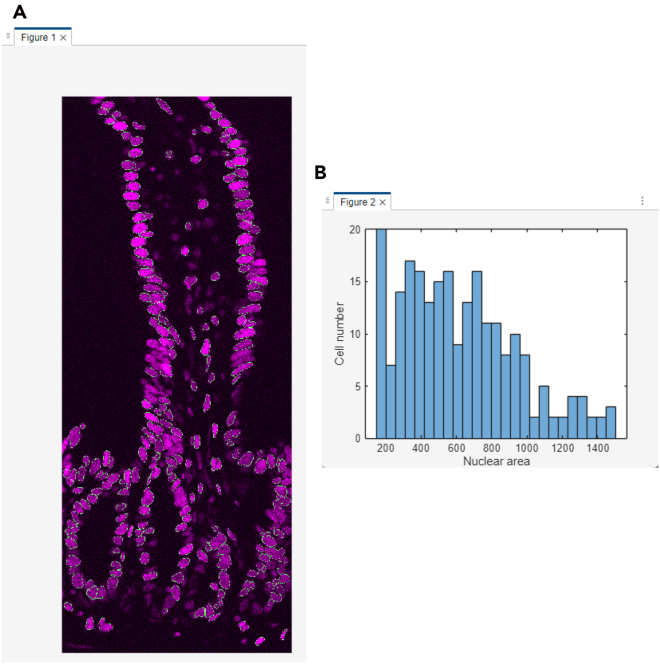
Debug windows for nuclear segmentation in the MATLAB-based image processing pipeline (A) A nuclear image displayed in magenta, overlaid with green nuclear mask contours. (B) A histogram of nuclear area from the primaryMaskRound.

***Note:****Nuclear segmentation* (Figure 4; debug point 1-1): nuclear images will be displayed in magenta, overlaid with green nuclear mask contours, for each 4i round (Figure 6A). Ensure that each nucleus is accurately segmented in the images; if not, click Stop and adjust the nuclear segmentation parameters in run_cryo4i.m. Additionally, a histogram of nuclear area from the primaryMaskRound will also be shown. Confirm that the histogram distribution appropriately covers the range of nuclear areas present in the image (Figure 6B); if not, click Stop and modify the debrisarea and boulderarea parameters in run_cryo4i.m.

Troubleshooting 5.

**Figure 7:**
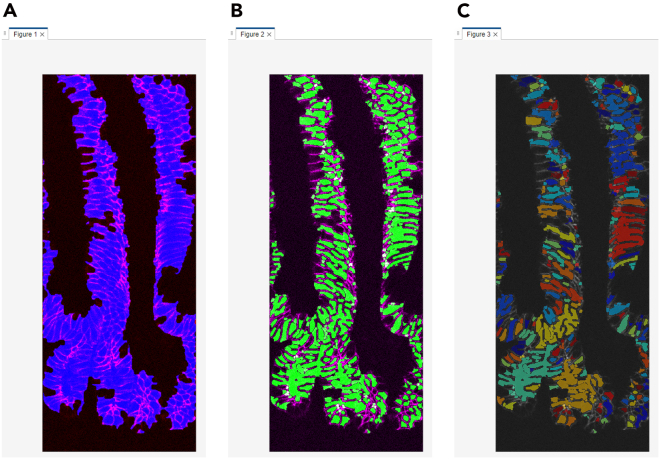
Debug windows for E-cadherin-based segmentation in the MATLAB-based image processing pipeline (A) An E-cadherin image displayed in magenta, overlaid with the blue E-cadherin positive cell area used for the E-cadherin positive/negative binary classification. (B) An E-cadherin image displayed in magenta, overlaid with the green E-cadherin mask used for cell segmentation. (C) An E-cadherin image displayed in white, overlaid with the E-cadherin masks for individual cell segmentation shown in distinct rainbow colors according to cell ID.

***Note:****(Optional) E-cadherin-based segmentation* (Figure 4; debug point 1–2): a set of three images will be displayed for each 4i round containing E-cadherin images. In the displayed images, the raw E-cadherin signal will appear in magenta or white. In the first image, the entire E-cadherin positive cell area will be shown in blue (Figure 7A). Ensure that the blue region specifically corresponds to E-cadherin positive cells when using the 'Nuc-Ecad boundary' method and the E-cadherin positive/negative binary classification. In the second image, the E-cadherin mask used for cell segmentation will be shown in green (Figure 7B). In the third image, E-cadherin masks for individual segmented cells will be displayed in distinct rainbow colors according to cell ID (Figure 7C). If using the ‘Ecad boundary’ method, verify that each cell is correctly segmented. If anything above is unsatisfactory, click Stop and modify the E-cadherin related parameters in run_cryo4i.m.

**Figure 8:**
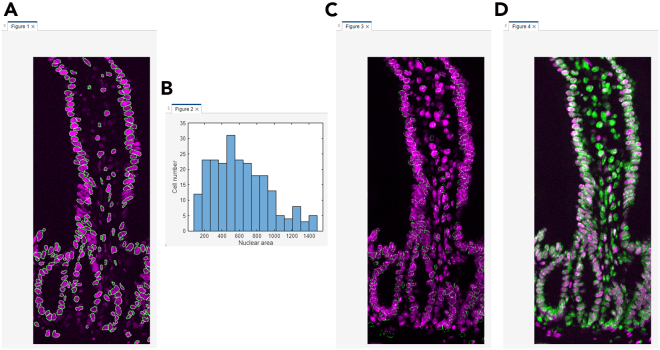
Debug windows for jitter alignment across 4i rounds in the MATLAB-based image processing pipeline (A) A nuclear image from the primaryMaskRound displayed in magenta, overlaid with green nuclear mask contours, after jitter alignment and cropping. (B) A histogram of nuclear area from the primaryMaskRound, after jitter alignment and cropping. (C) A nuclear image from the different round of 4i displayed in magenta, overlaid with green nuclear mask contours from the primaryMaskRound, after jitter alignment and cropping. (D) An overlaid images of the nuclear image from the Nth (magenta) and (N+1) th (green) rounds of 4i, after jitter alignment and cropping.

***Note:****Jitter alignment across 4i rounds* (Figure 4; debug point 2): The displayed images have been processed through jitter alignment and cropping. In the first image, the nuclear image from the primaryMaskRound will be displayed in magenta, overlaid with green nuclear mask contours (Figure 8A). In the second image, a histogram of nuclear area will also be shown (Figure 8B). The next set of images are nuclear images for each 4i round in magenta, overlaid with the green nuclear mask contours from the primaryMaskRound (Figure 8C). The last set of images are the overlaid images of the nuclear image from the Nth (magenta) and (N+1) th (green) rounds of 4i (Figure 8D). Most nuclei should appear white, indicating successful jitter alignment. If anything above is unsatisfactory or the error message ‘Max jitter too high’ appears in the command window, click Stop and verify the 4i images represent the same field of view with an identical sets of cells, and test alternative jitterIndex channel in run_cryo4i.m.

**Figure 9:**
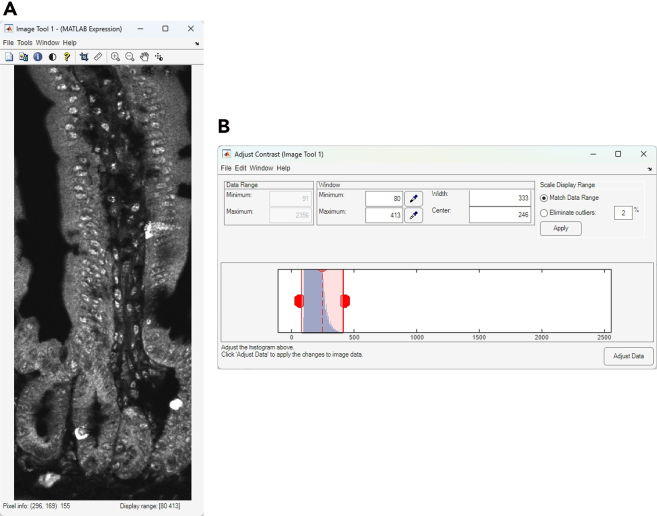
Debug windows for puncta threshold determination in the MATLAB-based image processing pipeline (A) An image used for puncta quantification displayed in gray in a MATLAB Image Tool window. (B) An Adjust Contrast window used for puncta threshold determination.

***Note:****(Optional) Puncta threshold determination* (Figure 4; debug point 3): images in grayscale for puncta quantification will be displayed (Figure 9A). Use the Adjust Contrast tool to estimate the average background intensity (Figure 9B), which will be removed during thresholding, assuming puncta are brighter than the background. Based on the background intensity, set the value of punctaThresh in run_cryo4i.m.

**Figure 10:**
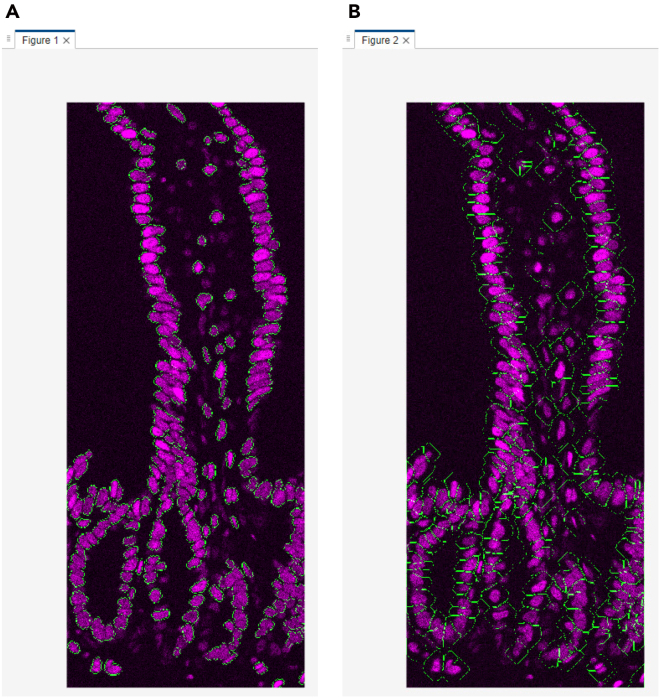
Debug windows for cytoring generation and cytoplasm mask for puncta quantification (A) A nuclear image displayed in magenta, overlaid with green cytoring contours. (B) A nuclear image displayed in magenta, overlaid with green cytoplasmic contours.

***Note:****(Optional) Cytoring generation* (Figure 4; debug point 4-1): nuclear images will be displayed in magenta, overlaid with green cytoring contours, for each 4i round that includes cytoring calculation (Figure 10A). Ensure that each cytoring is appropriately sized to cover the cytoplasmic signal; if not, click Stop and adjust outerrad_cytoring and outerrad_cytoring in run_cryo4i.m.

***Note:****(Optional) Cytoplasm mask for puncta quantification* (Figure 4; debug point 4-2): image of the first channel from the first round of 4i will be displayed in magenta, overlaid with green cytoplasmic contours (Figure 10B). Ensure that each cytoplasmic mask is appropriately sized to cover the puncta signal in the cytoplasm; if not, click Stop and modify the thickenradius parameter in run_cryo4i.m.

50. After image processing is completed, confirm that the elapsed time will be displayed in the command window.

***Note:*** The output files, including IHCdata_1.mat, settings_IHC.mat, and the processed images (cropped and mask images), will be saved automatically. IHCdata_1.mat contains quantified data, with each cell represented in a separate row and each parameter in a separate column. settings_IHC.mat contains ‘settings’ used in run_cryo4i.m and ‘header’. The ‘header’ corresponds to the parameters saved in IHCdata_1.mat: ‘x’ and ‘y’ represent the X and Y coordinates of each nuclear centroid, and ‘nuclear area’ indicates the number of pixels in each nuclear mask. These are followed by signal intensity values for each channel from each 4i round, and an E-cadherin positive/negative classification.

51. After a final round of quality check, proceed to batch analysis if required.
  ***Optional:****Batch analysis*: Once the image analysis parameters have been optimized for the first site (field of view), perform batch analysis across multiple sites using the same parameters. Ensure that the images for each site are saved in the corresponding 4i round folders, for example:

Round1/Example_1.tif, Example_2.tif

Round2/Example_1.tif, Example_2.tif

Round3/Example_1.tif, Example_2.tif

Execute the following command in the MATLAB command window.

>sites = 1:2;

>Parallelsite({@Fixed_IF_Ecad},settings,sites,0)

52. Load the output files including IHCdata_1.mat and settings_IHC.mat into the workspace by using load in the command window or dragging and dropping the files into the workspace.
53. To plot the intensity of the protein of interest (POI), first set the input parameters in the MATLAB script scatterplot_POI.m.

***Note:*** The parameters are described as follows:

pixelsize: the pixel size, the physical dimensions of a single pixel in microscopy images, typically measured in micrometers (μm).

POI_formula: Depending on the signal type in each channel, choose the appropriate quantification formula method below:

‘nuc’ – Signal intensity quantification in the nucleus, as defined by ‘POI_nuc_col’.

‘cyto’ – Signal intensity quantification in the cytoring, as defined by ‘POI_cyto_col’.

‘nuc-cyto’ – Signal intensity quantification in the nucleus minus cytoring, as defined by ‘POI_nuc_col’-‘POI_cyto_col’.

‘cyto-nuc’ – Signal intensity quantification in the cytoring minus nucleus, as defined by ‘POI_cyto_col’-‘POI_nuc_col’.

POI_nuc_col: A column number used for quantification in the nucleus. Refer to the header in settings_IHC.mat to select the appropriate column.

POI_cyto_col: A column number used for quantification in the cytoplasm. Refer to the header in settings_IHC.mat to select the appropriate column.

54. Execute the following command in the MATLAB command window:

>scatterplot_POI(IHCdata)

***Note:*** Two figures will be displayed. The first figure shows the quantification of the microscopy image color-coded by POI intensity (Figure 5; left). The second figure shows the quantification of POI intensity as a function of cell position along the crypt-villus axis (corresponding to Y (μm) in the first figure), with a red mean + standard deviation (SD) line per 20 μm-sized X axis bin (Figure 5; right).

## Expected outcomes

Cryo-4i is a powerful method for obtaining multiple protein signals from the same tissue sample. Compared to other multiplexed tissue imaging methods, Cryo-4i offers the advantages of low cost and accessibility, as it relies on standard reagents and does not require specialized equipment. Using mouse small intestinal epithelium as a model system, we successfully detected ten different markers, Lgr5, phospho-Rb, phospho-SMAD1/5/8, Vimentin, Mucin 2, Aldolase B, E-cadherin, Histone H2B, Histone H3, and Hoechst, across five rounds of Cryo-4i in the same tissue (Figures 1A–1F). To validate antibody elution efficiency, we performed the standard Cryo-4i protocol with a negative control in which the tissue was re-stained with only the secondary antibody in the second round, omitting the primary antibody. In this condition, no detectable signal from the primary antibody used in the first round was observed, confirming sufficient antibody elution (Figure 2A). We also validated the consistency of tissue staining across multiple Cryo-4i rounds by repeatedly staining for Mucin 2 over five cycles (Figure 3A). The positive and negative concordance rates ranged from 76.9% to 100%, confirming the reproducibility of staining throughout the process (Figure 3B). We note that changes in the signal-to-noise ratio were observed across different staining rounds, likely due to the effect of the elution buffer, which could also act similarly to an antigen retrieval treatment. In consistent with this observation, a previous report suggested that antibody elution by incubation at 66°C in 2-mercaptoethanol and sodium dodecyl sulfate (2-ME/SDS) may also have an antigen-retrieving effect.^15^

In combination with our MATLAB-based automated image analysis pipeline (Figure 4), we quantified protein expression at the single-cell level. As expected, (1) the stem cell marker Lgr5 was enriched at the crypt base, where intestinal stem cells reside^16^; (2) the proliferative marker phospho-Rb was elevated in the upper crypt region, corresponding to the location of transit-amplifying cells with high proliferative capacity^17^^,^^18^; (3) the BMP signaling readout phospho-SMAD1/5/8 was higher in the villus, consistent with prior studies demonstrating a BMP gradient along the crypt–villus axis^19^^,^^20^ (Figure 5).

In summary, the Cryo-4i protocol provides a robust and comprehensive approach for spatially resolved, multiplexed protein profiling in tissue sections. It enables detailed characterization of protein expression patterns and post-translational modifications within their native tissue context.

## Limitations

One of the limitations of the Cryo-4i protocol is the risk of tissue architecture damage across multiple rounds of 4i, particularly during the elution step. This step employs an elution buffer containing protein reductants (TCEP), denaturants (urea and guanidine hydrochloride), which can compromise tissue integrity. While the overall tissue architecture was largely preserved, careful quantification indicated that some cells detected in the first round were no longer detectable by the fifth round. Therefore, careful selection of regions of interest will be necessary when analyzing these tissues. Additionally, the antigenicity of sensitive antigens and nuclear DNA staining may diminish with successive rounds of 4i. On a side note, the elution buffer quenches fluorescence likely due to the reducing agent TCEP (Figure 2B); therefore, tissues expressing fluorescent proteins (e.g., from reporter mice) should be imaged in the first round of 4i. Similarly, nuclear staining becomes less distinct through the 4i cycles, complicating single-cell segmentation. To address this issue, we recommend staining cell boundary markers (e.g., cadherins such as E-cadherin, catenins such as β-catenin and integrins) in combination with nuclear markers in all 4i rounds. Although our experiments confirmed that valid data can be collected after 5 iterations, the original 4i protocol recommends a maximum of 25 iterations and the maximum number of iterations for Cryo-4i is yet to be determined. Another limitation of the Cryo-4i protocol is that it requires a longer processing time than the original 4i protocol, as primary antibody incubation often needs to be performed overnight (∼16 h) rather than for 2 h. This is likely due to differences between tissue sections and cultured cells.

## Troubleshooting

### Problem 1

Tissue detachment from glass slides (related to step 16).

### Potential solution

Tissue detachment from glass slides can occur for several reasons, including inadequate slide coating, insufficient drying of the slides, poor adhesion of unfixed tissue to the glass surface, or preparation of cryosections that are too thick. To address these issues, it is important to use adhesive-coated slides and confirm that they are within their expiration date. Allowing the sections to air-dry for a longer period ensures that residual moisture is completely removed, which improves adhesion. In addition, post-fixing cryosections on the slides with PFA can further enhance tissue attachment. Finally, preparing cryosections at an optimal thickness of 5–10 μm helps to minimize detachment and preserve tissue morphology.

### Problem 2

Weak or no signal in tissue staining in the first round of 4i (related to step 27).

### Potential solution

Weak or absent signal in tissue staining during the first round of 4i may result from antigen masking, suboptimal antibody concentration, poor antibody quality, inadequate permeabilization, or over-fixation of the tissue. To overcome these issues, antigen retrieval can be performed using either heat-induced or enzymatic methods to expose masked epitopes. Adjusting the primary antibody concentration or extending the incubation time may improve staining intensity. It is also important to use fresh, validated antibodies obtained from reliable sources to ensure specificity and sensitivity. Optimizing the permeabilization step, for example by modifying the detergent type or concentration, can enhance antibody access to intracellular targets. Finally, reducing fixation time or concentration, or using a milder fixative, helps to prevent over-fixation that may hinder antigen recognition.

### Problem 3

Residual staining from the previous primary antibody (related to step 29).

### Potential solution

Residual staining from the previous primary antibody may arise due to insufficient antibody elution. This issue can be resolved by applying harsher elution conditions, such as increasing the concentration of protein reductant (TCEP) and denaturants (urea and guanidine hydrochloride), adding another reductant (e.g., 2-mercaptoethanol), raising the reaction temperature (e.g., 55°C), or extending the incubation time to ensure more thorough removal of the bound antibody.

### Problem 4

Weak or no signal from the subsequent rounds (related to step 29).

### Potential solution

Weak or absent signal in the subsequent rounds of 4i can occur due to loss of antigenicity during earlier elution steps. To address this problem, milder elution conditions should be applied, such as reducing the concentration of protein reductants and denaturants, lowering the reaction temperature, or shortening the incubation time. In addition, the order of antibody application can be optimized by assigning antibodies that perform less robustly to the earlier rounds, while reserving higher-performing antibodies for later rounds to maintain signal quality throughout the experiment.

### Problem 5

Inaccurate nuclear segmentation (related to step 49).

### Potential solution

Inaccurate nuclear segmentation can result from a low signal-to-noise ratio in the nuclear stain, suboptimal nuclear detection, or the presence of concatenated nuclei. To improve segmentation accuracy, the quality of the nuclear stain should be enhanced and image acquisition settings such as exposure time and gain can be adjusted. Alternative nuclear segmentation methods can also be tested by modifying parameters such as segmethod and adjusting the nuclear radius parameter nucr. In cases where nuclei appear concatenated, performing E-cadherin staining and applying the “Nuc-Ecad boundary” segmentation method can help to separate overlapping nuclei more effectively.

## Resource availability

### Lead contact

Further information and requests for resources and reagents should be directed to and will be fulfilled by the lead contact, Yumi Konagaya (yumi.konagaya@riken.jp).

### Technical contact

Technical questions on executing this protocol should be directed to and will be answered by the technical contact, Shuji Matsuguchi (shuji.matsuguchi@riken.jp).

### Materials availability

The R26-H2B-mScarlet mice (RIKEN LARGE strain: CDB408E; https://large.riken.jp/distribution/reporter-mouse.html), which ubiquitously expresses H2B-mScarlet-HA, were established from the conditional R26R-H2B-mScarlet mice (RIKEN LARGE strain: CDB407E; https://large.riken.jp/distribution/reporter-mouse.html) by removing the loxP-flanked STOP cassette via Cre recombinase. The R26R-H2B-mScarlet mice were generated by CRISPR/Cas9-mediated genome editing in zygotes as previously described.^21^ The cDNA encoding H2B-mScarlet-HA-WPRE, derived from pmScarlet-i_C1^22^ (Addgene plasmid: #85044) was cloned into a modified pROSA26-STOP-DEST vector using the gateway system (Invitrogen). Routine genotyping PCR was performed using primers as previously described.^23^ We used three kinds of transgenic male mice: (1) R26-H2B-mScarlet, (2) LGR5-eGFP-IRES-CreERT2^16^ (The Jackson Laboratory strain: #008875), and (3) R26-H2B-mScarlet; LGR5-eGFP-IRES-CreERT2.

### Data and code availability

• The accession number for the image data reported in this paper is SSBD: https://doi.org/10.24631/ssbd.repos.2026.02.491.
• The accession number for the code reported in this paper is GitHub: https://github.com/Yumi-Konagaya-Lab/cryo-4i-image-analysis-matsuguchi-2025.git.

## Acknowledgments

We thank all members of the Konagaya Laboratory and the Morimoto Laboratory for their helpful input. In particular, we are grateful to Naomi Helga Iizuka for her valuable contributions to image analysis, and to Miki Fujiwara for her kind assistance. The imaging experiments were performed at the Kobe BioImaging Facility and Factory at RIKEN BDR. The ECCD2 hybridoma was provided by the RIKEN BRC through the National Bioresource Project of the MEXT/AMED, Japan; we thank the staff for their support. LGR5-eGFP-IRES-CreERT2 mice^16^ were originally obtained from The Jackson Laboratory (strain #008875) and were kindly provided by Dr. Takashi Tsuji (formerly RIKEN BDR; currently at OrganTech, Inc., Japan). This work was supported by the Japan Society for the Promotion of Science (JSPS) KAKENHI, grant no. JP24K18118 (to Y.K.), and the Japan Science and Technology Agency (JST) as part of Adopting Sustainable Partnerships for Innovative Research Ecosystem (ASPIRE), grant no. JPMJAP24B1 (to Y.K.).

## Author contributions

S.M. developed the Cryo-4i protocol and performed all the experiments for the initial manuscript submission. S.M., A.Y., A.O., and Y.K. contributed to the experiments conducted during the revision. A.O. assisted with the experiments and mouse colony maintenance. T.A. and H.K. generated and established R26-H2B-mScarlet transgenic mouse line. Y.K. developed the MATLAB-based image analysis pipeline. S.M. performed trial-and-error testing with the pipeline to analyze the data. S.M. and Y.K. designed the project. S.M., A.Y., and Y.K. wrote the manuscript.

## Declaration of interests

The authors declare no competing interests.
