# Supplementary material for: Protocol for iterative indirect immunofluorescence imaging of frozen mouse intestinal tissues
Source: STAR Protoc. 2026 Mar 7;7(1):104410. doi: 10.1016/j.xpro.2026.104410 (PMC12992094; doi:10.1016/j.xpro.2026.104410)
Supplement: Document S1. Figure S1 and Methods S1 [file mmc1.pdf]

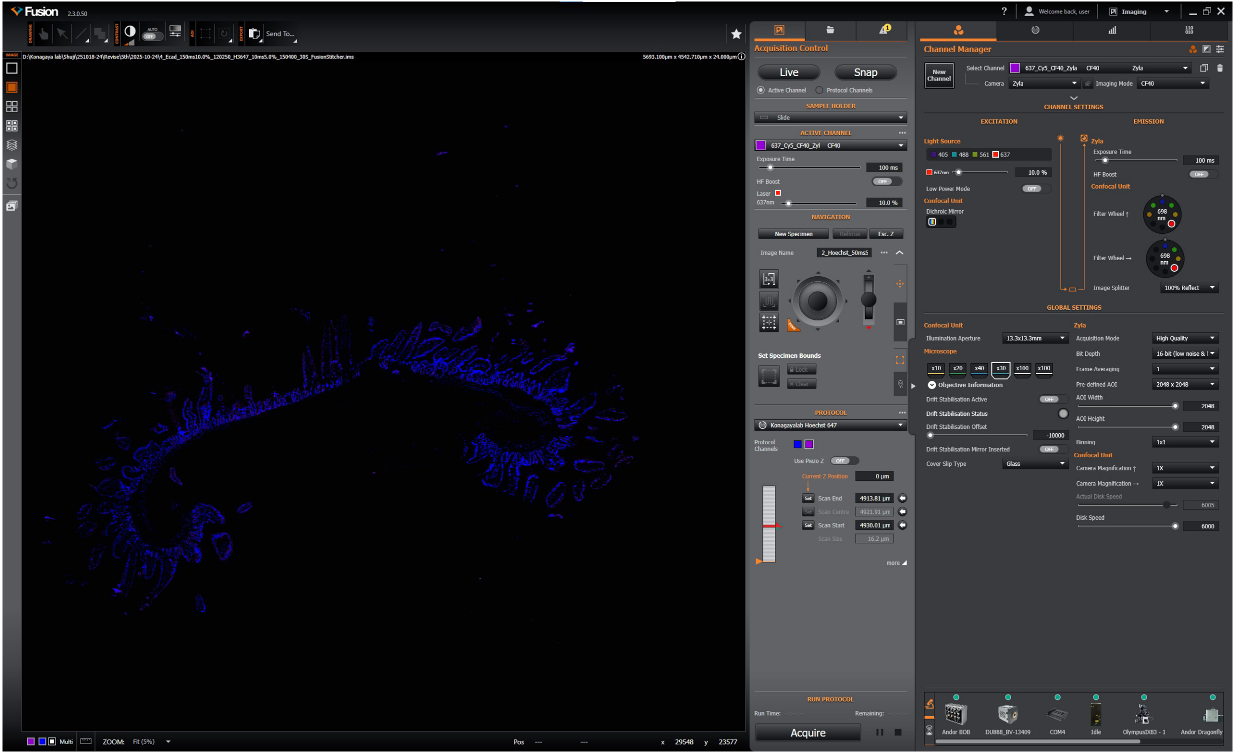

Figure S1: Image acquisition settings in Fusion software, related to Step 27.

Methods S1: Microscopy metadata provided according to the Microscopy Metadata Checklist template, related to Step 27.

Microscopy Metadata Checklist

*** Asterisks indicate optional items

Microscope Stand and Motorized Components

- ☐ Microscope Stand manufacturer and Model *Andor, Dragonfly202; Olympus (Evident), IX83-ZDC*
- ☐ Illumination Shutter Manufacturer and Model *Andor, LC-ILE-400-M*
- ☐ Stage Manufacturer and Model *ASI, Automated Stage with Piezo Z-Axis Top Plate*
- ☐ Linear encoded stage? *Linear encoded stage*
- ☐ Focusing device manufacturer and model *ASI, Piezo-actuated drive; Olympus (Evident), IX83 Z drive*
- ☐ Focusing device type *Nosepiece, piezo*
- ☐ Hardware-based Focus maintenance device manufacturer and model *Olympus (Evident), IX3-ZDC2 (Z-Drift Compensation)*
- ☐ So˝ware based focusing maintenance set up (wavelength, range, step size, algorithm) *Andor, Fusion software*
- ☐ Excitation/Emission filter wheel Manufacturer and Model *Andor, CR-DFLY-CAM-WHL*
- ☐ Excitation/Emission filter wheel Location in the Lightpath *in front of the camera*

- ☐ *** Type *Compound*
- ☐ *** Commercial/commercial modified, custom modified *N/A*
- ☐ *** Upright or inverted *Inverted*
- ☐ *** Illumination Shutter speed
- ☐ *** Stage precision and speed
- ☐ *** Focusing device range
- ☐ *** Focusing device speed
- ☐ *** Focusing device precision
- ☐ *** Excitation/Emission filter wheel filter change speed

Spinning Disk Confocal-Specific Hardware and Settings

- ☐ Scan unit Manufacturer and Model *Andor Dragonfly202*
- ☐ Pinhole size *40 um*
- ☐ *** Pinhole spacing
- ☐ *** Number of disks *Micro-lens enhanced dual disk*
- ☐ *** Disk speed *400 fps*

Illumination

- ☐ Laser launch/combiner manufacturer and model *Andor, Borealis™ enhanced illumination System*
- ☐ Laser line/wavelength *405 nm; 488 nm; 561 nm; 637 nm*
- ☐ Laser type *Solid State*
- ☐ Laser modulation *Directly modulated*
- ☐ *** Laser power output *100 mW (405 nm); 150 mW (488 nm); 100 mW (561 nm); 140 mW (637 nm)*

Wavelength Selection

- ☐ Filter manufacturer and product number *Semrock LED-DA/FI/TR/Cy5/Cy7*
- ☐ Filter center wavelength and bandwidth (FWHM), cut on or cut off wavelength *445 /46 nm, 521/38 nm, 594/37 nm, 698/77 nm, 525/50 nm, 600/50 nm*
- ☐ Filter coating method *Hard splutttered; soft coated*
- ☐ *** Additional filters manufacturer and model
- ☐ *** If tunable wavelength selection, range of wavelengths detected

Optics

- ☐ Objective manufacturer *Olympus (Evident)*
- ☐ Objective correction *UPLSAPO (Universal Plan Super Apochromat)*
- ☐ Objective magnification *30X*
- ☐ Objective numerical aperture *1.05 N.A.*
- ☐ Specified immersion medium *Silicone Oil*
- ☐ *** Objective application *Fluorescence (Visible/NIR)*
- ☐ *** Immersion medium manufacturer and product number (if used) *Olympus SIL300CS-30CC*

Detection

- ☐ Camera manufacturer and model *Andor Zyla Z4.2P-USB3*
- ☐ Camera type *sCMOS*
- ☐ Binning *1x1 (no binning)*
- ☐ Bit Depth and associated gain *16 bit*
- ☐ EM gain (if EMCCD used) *N/A*
- ☐ *** Pixel size *6.5 x 6.5 um*
- ☐ *** Chip size *2048 x 2048 pixels*
- ☐ *** Pixel readout rate *216 MHz (108 MHz×2 sensor halves); 540 MHz (270 MHz×2 sensor halves)*
- ☐ *** Dynamic range *33,000 : 1*
- ☐ *** Readout noise *0.90 e- [1.1 e-]@216 MHz , rolling shutter; 1.10 e- [1.3 e-]@540 MHz , rolling shutter*
- ☐ *** If sCMOS, rolling or global shutter *Rolling and global*

Acquisition So˝ware

- ☐ So˝ware manufacturer, name, and version *Fusion v. 2.3.0.50*
- ☐ If custom, Author and appropiate citation *N/A*
- ☐ State of the shutter during acquisition *Shutter was open during acquisition*
- ☐ Order of experimental acquisition *First, open shutter and a Z-stack was captured, close shutter, then moved to next x,y stage position, open shutter, trigger camera, collect next Z-stack*
- ☐ If custom macro: cite or make available. *N/A*
- ☐ Sequential, simultaneous, or triggered channel/color acquisition *Images were acquired sequentially*

- ☐ Zstack focusing device *ASI, Automated Stage with Piezo Z-Axis Top Plate*

☐ Zstack range and step size *10-30 um range 1-2 um step size*
☐ *** Specific aquisition Modules/Macros/apps *Fusion custom Protocol*

Sample Preparation

☐ Sample holder type, manufacturer and product number *ASI, I-3091 Universal Insert for piezo top plates*

☐ Coverslip grade *#1.0 (0.13–0.17 mm)*
☐ Coverslip coating (type, concentration, detailed protocol) *N/A*
☐ Detail protocol: fixitive, concentration of fixitive, fixation conditions (bu˙ers, time, temperature), blocking, binding and hybridization bu˙er composition, Ab manufacturer, lot number, concentration, probe concentration, binding or hybridization conditions (time, temperature, sequential/simultaneous) *Refer to the main text*
☐ Mounting/imaging medium name, manufacturer and product number *50% glycerol (vol/vol) in PBS*

☐ Specific fluorescent protein variant *eGFP, mScarlet*
☐ Organic dye name, manufacturer and product number *Hoechst 33342, Thermo Fisher, Cat#H3570*
☐ Organic dye concentration and solvent *2 ug/mL*
☐ *** Mounting/imaging medium RI if not specified and curing time. *1.4 refractive index; 30 min curing time*

Acknowledgements

☐ Acknowledge the core facilities that facilitated your research *The authors gratefully acknowledge the Kobe BioImaging Facility and Factory (KBiIF) Core for their support and assistance in this work.*
☐ *** Check if specific equipment grants that funded the core facilities also need to be acknowledged (e.g., S10 Shared equipment grants) *N/A*

For more information, check out our paper: Montero-Llopis et al., 2021 (citation)

Microscopy Metadata Checklist Generator (MicCheck) developed by Rebecca Sen˝ (2021)
